# Species Associations in a Species-Rich Subtropical Forest Were Not Well-Explained by Stochastic Geometry of Biodiversity

**DOI:** 10.1371/journal.pone.0097300

**Published:** 2014-05-13

**Authors:** Qinggang Wang, Dachuan Bao, Yili Guo, Junmeng Lu, Zhijun Lu, Yaozhan Xu, Kuihan Zhang, Haibo Liu, Hongjie Meng, Mingxi Jiang, Xiujuan Qiao, Handong Huang

**Affiliations:** 1 Key Laboratory of Aquatic Botany and Watershed Ecology, Wuhan Botanical Garden, Chinese Academy of Sciences, Wuhan, Hubei, PR China; 2 Life Science College, University of Chinese Academy of Sciences, Beijing, PR China; Cirad, France

## Abstract

The stochastic dilution hypothesis has been proposed to explain species coexistence in species-rich communities. The relative importance of the stochastic dilution effects with respect to other effects such as competition and habitat filtering required to be tested. In this study, using data from a 25-ha species-rich subtropical forest plot with a strong topographic structure at Badagongshan in central China, we analyzed overall species associations and fine-scale species interactions between 2,550 species pairs. The result showed that: (1) the proportion of segregation in overall species association analysis at 2 m neighborhood in this plot followed the prediction of the stochastic dilution hypothesis that segregations should decrease with species richness but that at 10 m neighborhood was higher than the prediction. (2) The proportion of no association type was lower than the expectation of stochastic dilution hypothesis. (3) Fine-scale species interaction analyses using Heterogeneous Poisson processes as null models revealed a high proportion (47%) of significant species effects. However, the assumption of separation of scale of this method was not fully met in this plot with a strong fine-scale topographic structure. We also found that for species within the same families, fine-scale positive species interactions occurred more frequently and negative ones occurred less frequently than expected by chance. These results suggested effects of environmental filtering other than species interaction in this forest. (4) We also found that arbor species showed a much higher proportion of significant fine-scale species interactions (66%) than shrub species (18%). We concluded that the stochastic dilution hypothesis only be partly supported and environmental filtering left discernible spatial signals in the spatial associations between species in this species-rich subtropical forest with a strong topographic structure.

## Introduction

One of the long-standing goals in community ecology is to understand how communities of plants are assembled [1]–[3]. There is increasing awareness of how spatial patterns and processes influence species coexistence. For example, the analyses of interspecific spatial associations between species pairs could contribute to a better understanding of plant species assemblages [4]–[8]. Three families of rules which are contrasting, but not necessarily mutually exclusive have been proposed to explain species associations among species. The biotic assemble rule emphasizes the importance of competition and assumes that species with similar ecological requirement should be less likely to coexist [9]–[11]. In the framework of competition theory, Janzen-Connell hypothesis [12], [13] which was proposed to explain species coexistence in species-rich forest communities, suggests that tree species in forests tend to be segregated with their parent trees, conspecific trees and even closely related trees [14]. In addition, it is not hard to deduce from Janzen-Connell hypothesis that heterospecific neighbors, especially distantly related neighbors can promote species coexistence (i.e., species herd prorection hypothesis) [15]. Therefore, the competition-based theory predicts more negative species associations for closely related species than expected by chance and/or more positive associations for distantly related species [16], although recent coexistence theory shows that the situation is more complex [17]. In contrast to the biotic rule, the abiotic assemble rule suggests that environmental filtering processes [18], [19] are important drivers for species assemblages, and species with similar ecological requirement can coexist in a given habitat. In terms of phylogeny or taxonomy, it predicts that closely related species should be more likely to be positively associated than expected and/or distantly related species would be more negatively associated [20], [21].

Basically both the biotic assemble rule and the abiotic assemble rule rely on the priori assumption that species with respect to persistence within communities are non-equivalent. Distinctly, neutral theory presumes that individuals are ecologically equivalent. For example diversity pattern in species-rich communities such as tropical forests can be well explained by neutral theory which assumes that species are functionally equivalent, and that the community can be well assembled by demographic stochasticity and dispersal limitation [22]. Similarly, the recent proposed assertion of independent species spatial arrangement for stochastic geometry theory of biodiversity [23] assumes that individuals of different species are placed independently. Wiegand et al. [24] compared the proportion of overall species associations and species interactions across three forest dynamic plots [25-ha Changbaishan (CBS) plot with 51 species, China; 25-ha Sinharaja plot with 205 species, Sri Lanka; 50-ha Barro Colorado Island (BCI) plot with 303 species, Panama]. They concluded that stochastic dilution effects in species rich communities could overpower deterministic signals of species interactions and lead to approximate independence in species placement. Many previous studies were seemly compatible with such stochastic dilution effects. For example, it has been suggested that the low probability of two different species encountering each other in species-rich communities could simply result in weak interactions between species pairs [25], [26]. Studies conducted at species-rich communities showed that the number of observed nearest-neighbor species combinations was much lower than the possible species pairs defined by observed list of species [27], [28] and only a few of species pairs showed detectable species associations [6], [24], [29]. Perry et al. [29] found evidence that supported such dilution effects in four species-rich shrubland communities in southwestern Australia. Nevertheless, the 55.6% significant associations (29.5% positive associations and 26.1 negative associations) at 0–5 m scale for the 20 adult dominant species in a tropical forest (Xishuangbanna, China) also have been reported [30]. Therefore, additional tests should be conducted in different plots to investigate whether such stochastic dilution hypothesis is a pervasive rule [24] in species-rich communities.

As sessile organisms, individuals of plants have their own dependence on local growing conditions and they interact locally [31]. Different mechanisms should leave a signature in the spatial pattern [6], [21]. Therefore, spatially explicit and individual-based point pattern analysis is ideally suited to evaluate the evidence for the interspecific associations among species in plant community. In this study, using the techniques of spatial point pattern analysis we quantified the interspecific associations among 51 dominant species in a fully-mapped 25-ha evergreen and deciduous mixed Forest Dynamic Plot (FDP) at Badagongshan (BDGS) in central China. The general objective of this study is to analyze species associations to find out the relative importance of stochastic dilution processes, environmental filtering and competition in this species-rich forest. To compare the results of BDGS plot with those of the 25-ha CBS plot, 25-ha Sinharaja plot and 50-ha BCI plot [24], we used the same method presented in Wiegand et al. [24]. We first analyze the overall spatial associations which could be potentially caused by environmental filtering and specie interactions (analysis 1). Then we selectively tested the fine-scale species interactions after controlling the large-scale environment effects (analysis 2). We proposed three specific hypotheses. If the stochastic dilution hypothesis was true, the proportion of no association type and segregation type in analysis 1 in BDGS plot should follow the tendency that the proportion of no association type increased with species richness (hypothesis H1) and that the proportion of segregation types decreased with species richness (hypothesis H2). If the stochastic dilution hypothesis was supported, the proportion of the significant species interactions in BDGS plot should be low (Hypothesis H3). However, if presence of strong species interactions and positive ones occur less frequently and the negative cases occur more frequently within the same species family, it would support the biotic assemble rule. Compared with CBS plot with much lower species richness and BCI plot with much higher species richness, we expected that dilution effects at BDGS plot should be stronger than CBS plot and weaker than BCI plot. Compared with Sinharaja plot, we expected that dilution effects at BDGS plot could be overpowered by environmental filtering, because species richness at BDGS plot was only a little higher but the fine-scale topographic structuring at BDGS plot was much stronger.

## Methods

### Study Sites

The study area is situated in Badagongshan National Nature Reserve, Hunan Province (29°46.04′ N, l10°5.24′ E), in north of the Wuling Mountains, a northern boundary of mid-subtropical zone. It has north subtropical mountain humid monsoon climate. Annual rainfall averages 2,105.4 mm, with the maximum of 2,840.1 mm, which is comparable to rainforests. Average annual rainy days are 176 days. The mean temperature here ranges from 0.1°C in January to 22.8°C in July with an annual mean of 11.5°C. Such meteorological information was obtained at elevation of 1,13 m. The annual rainfall increases and the mean temperature decreases with elevation. The topography is characterized with deep valleys, steep slopes and flat tops. The main soil types are yellow-red soil (below 460 m), mountain yellow soil (460–1,000 m) and yellow-brown soil (above 1,000 m) [32]. BDGS is located at the eastern Sichuan-western Hubei endemic plant genus distribution center (relic center) in China’s endemic plant annular region which spans eastern Sichuan province, southwestern Hubei province, northwest Hunan province and northeastern Guizhou province. Primary forests are mainly distributed more than 1,000 m above sea level, and *Fagus lucida* and its mixed forests make up 60% of natural forest between the elevations of 1,200 and 1,890 m. Dominant deciduous trees include *F. lucida*, *Carpinus fargesii*, *Castanea seguinii*, *Sassafras tzumu*, *Sorbus folgneri, Davidia involucrate*, *Dendrobenthamia capitata*, and dominant evergreen trees include *Cyclobalanopsis multinervis, Camellia pitardii*, *Litsea elongate*, *Photinia beauverdiana*, and *Rhododendron sutchuenense*.

In 2011, we established the 25-ha (500 m×500 m) FDP at BDGS. All free-standing trees with DBH≥1 cm were tagged, mapped and identified to species following the plot standards of Center for Tropical Forest Science (CTFS) network [33]. We collected 238 species, 114 genera and 53 families in the first census of 2010–2011. The 25-ha BDGS plot is an evergreen and deciduous subtropical forest plot. The soil type is yellow-brown soil. The elevation in this plot ranges from 1,369.6 m to 1,470.9 m. The plot also shows strong fine-scale topographic structures with deep valleys and steep slopes (Figure S1). Mean annual precipitation is above 2,105 mm. The plot is old-growth forest without large scale disturbances. No specific permits were required for the described field studies. To make our study comparable with previous studies [6], [7], [24], we restrict our analysis to 51 species (Table S1) which have more than 70 individuals with Diameter at Breast Height (DBH) greater than 10 cm. Species were grouped into two life forms by the maximum attainable height: arbors (>5 m) and shrubs (<5 m).

### Statistical Analysis

#### Analysis 1: overall species associations

To quantify overall species associations and facilitate direct comparison of the results, we used the methodology presented in Wiegand et al [24], i.e., we employed a two-dimensional classification scheme based on two statistics K-function K_12_ (r) [34], [35] and the cumulative nearest neighbor distribution function *D*_12_ (r) [36] to categorize all the possible species pairs. The bivariate *K*-function *K*_12_ (r) is defined as the expected number of species 2 within radius r of an arbitrary species 1 point, divided by density λ_2_ of species 2 [34]. *D*_12_(r) gives the probability that the nearest 2 neighbor of an individual of species 1 is located within distance r. To distinguish the different types of species associations from those that may arise purely by chance, the Homogeneous Poisson process was implemented as a null model in which the locations of the focal species remained unchanged, but trees of species 2 were distributed randomly and independently of the locations of species 1. The significant effects detected by this null model can be due to (i) shared or opposed habitat associations of the species pair, (ii) species interactions, and (iii) univariate clustering of the two species. The expectation of the summary statistics under the null model yield 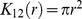 and 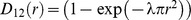. The two axes of the classification scheme were defined as
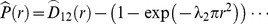
(1)

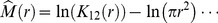
(2)


The hat symbol indicates the observed value. The theoretical values under the null model was subtracted to move the null expectation onto the origin of the scheme and log-transformed the *K*-function in order to weigh positive or negative departures from the null model in the same way [6], [24]. Following this two-axis scheme, the species associations can be grouped into four types: Type Ι: ‘segregation’ [M(r)<0 and P(r)<0] where individuals of species 2 occur on average less frequently with species 1 neighborhoods than expected by chance alone; type II: ‘partial overlap’ [M(r)>0 and P(r)<0], where some neighborhood of species 1 contain more individuals of species 2 and other less; type III: ‘mixing’ [M(r)>0 and P(r)>0], where individuals of species 2 occur on average more frequently within species 1 neighborhoods; and type IV: [M(r)<0 and P(r)>0], only arises if strong second-order effects occur. Species pairs that show non-significant effects for a given neighborhood r in both summary statistics are classified as ‘no association’ type and will be located closely to the origin of the scheme.

#### Analysis 2: fine-scale species interactions

To facilitate the direct comparison of results in different plots, we also employed the pair correlation function g_12_(r) [37] to assess the prevalence of fine-scale species interactions, which was presented in Wiegand et al. [24]. The g-function is related to the derivative of the *K*-function, i.e., d*K_1_*_2_(r) = g_12_(r)2πr dr, and λ_2_ g_12_(r) can be interpreted as the density of species 2 plants at distance (r−dr/2, r+dr/2) from species 1 plants, where dr is the “ring width”. We used the Heterogeneous Poisson process (HP) [38] as a null model to test the fit of each pair of species. The HPs were estimated non-parametrically for each pair via kernel density methods using an *Epachenikov* kernel [6]. The locations of the individuals of species 1 were kept unchanged and the locations of the individuals of the species 2 were randomized using its own intensity function under the HP model. Trees of species 2 were under this null model only locally displaced and the intensity function of this species (which was strongly influenced by habitat associations) was fixed for the null model. As a consequence, this null model can approximately factor out effects of the environment and therefore reveal potential effects of species interactions. However, an assumption of this method was that the habitat association of the species 2 varied over spatial scales larger than the bandwidth of the kernel estimate (i.e., separation of scales). If the assumption of separation of scales holds, the significant effects should disappear in less than 30 m (i.e., the bandwidth of the HP process) [6]. Previous studies suggested that the direct plant-plant interactions among larger trees occurred within 30 m [6], [7]. Thus a kernel bandwidth of 30 m was used in this study. The bivariate pair-correlation function g_12_(r) was used as the test statistic. All pairs of species, i.e., species 1 versus species 2 and species 2 versus species 1 were tested since we cannot assume that the associations would be symmetric.

### The Significant Test of the Point Process Models

We selected a spatial resolution of 2 m for all analyses, and a ring width of dr = 6 m for fine-scale bivariate analyses. This is a sufficiently fine resolution compared with dimensions of the 500 m×500 m plot and sufficiently fine to answer our questions. All these analyses were conducted with the *Programita* software [38], [39]. To avoid edge effects, all cases used the area-weighted edge corrections except *D*_12_ function [36], [38]. For the overall species associations, the empirical summary statistics were compared with those generated by 199 simulations of the homogeneous Poisson null model. The overall fit of the null model was assessed by the Goodness-of-Fit (GoF) test [40] which reduced the scale-dependent information contained in a summary statistic over 0−50 m into a single test statistic. We needed an error rate of 0.025 for each test statistic individually, because we used for the scheme two test statistics at the same time and to yield an overall 0.05 error rate if the two summary statistics are independent. If they are correlated we may have a lower error rate. For fine-scale species interactions, using the 199 simulations of the Heterogeneous Poisson null model, the GoF test was conducted with the pair correlation function over the 0−30 m distance interval with the 0.05 error rate.

To reveal the influence of univatiate species aggregation and species abundance on the significant fine-scale associations, we correlated the rank of Goodness of Fit test with the stem counts of the species pairs and the univariate g(r) of focal species or the neighbor species at scales r = 0, 6, 10, 20, 30 m using the Spearman rank test.

To find out whether species with shared families and fruit types showed more (or less) significantly positive (or negative) fine-scale species interactions than expected by chance, we run Permutation tests of association matrix (Table S2) under 10,000 randomization of significant cases over all pairs of species, following Wang et al. [7]. The number of observed significant cases and the number of significant cases under randomization per time were counted. If the number of observed significant cases located in the largest or smallest 0.025 of the 10,000 randomization, we consider it to be significant.

The correlation analyses and the Permutation tests were conducted in *R* software [41].

## Results

### Analysis 1: Overall Species Associations

**Figure 1:**
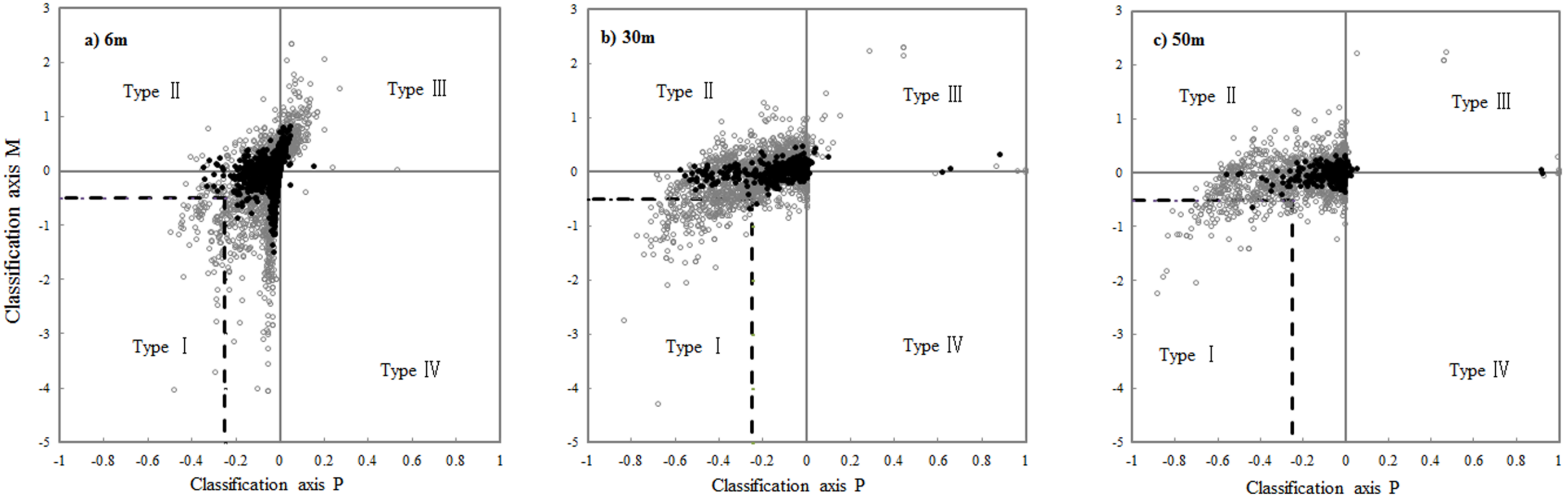
Classification of overall species associations at 6-, 30- and 50 -m scale. Allocation of overall species associations of 2,550 species pairs based on the classification axes defined in equation (1) and (2). The grey circles indicated the significant associations (i.e., significant departure from the null model by K_12_ and D_12_).

Figure 1-a, b and c showed the resulting association types of species pairs for 6 m, 30 m and 50 m neighborhoods at BDGS plot. To quantify the proportion of species pairs with strong segregation we defined all cases where P<−0.25 and M<0.5 (dashed lines in Figure 1) were strong segregations. We found 26.2, 21.1 and 16.4% of all species pairs at 6 m, 30 m and 50 m respectively at BDGS plot showed strong segregations. The proportion of the positive associations (i.e., mixing type III) was quite high for 6 m neighborhood analysis, but they completely disappeared at 30 m neighborhood (Figure 1).

**Figure 2:**
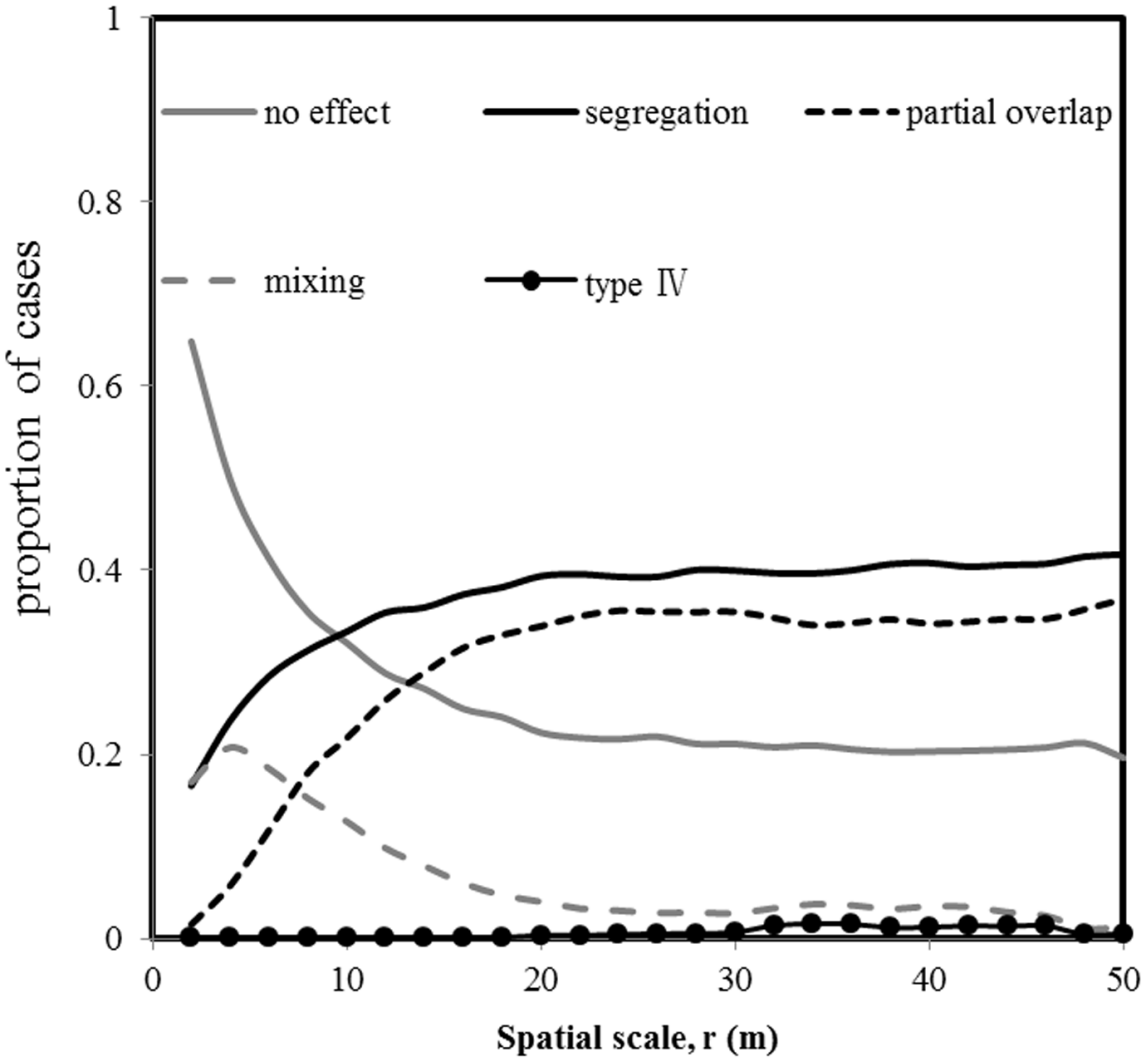
Overall interspecific association patterns in dependence on the scale r.

As Figure 2 showed, the proportion of the no association type decreased with neighborhood size. No association type governed the species association patterns within neighborhoods of 8 m. For neighborhood greater than 20 m, the BDGS plot showed a low proportion of the no association type of approximately 20%. The type of segregation and partial overlap increased and the proportion of mixing peaked at neighborhoods of approximately 20 m.

**Figure 3:**
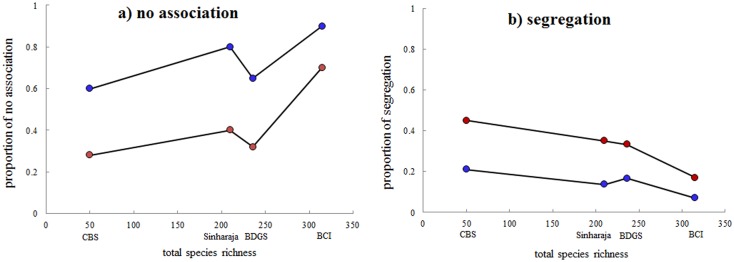
Relationship between richness and overall association patterns using the data from Badagongshan (BDGS) plot reported here and those reported in Wiegand et al. (2012) for Changbaishan (CBS) plot, Sinharaja plot and Barro Colorado Island (BCI) plot. (a) the proportion of no assciation type at 2 m (red circle) and 10 m neighborhood (blue circle); (b) the proportion of segregation type at 2 m (red circle) and 10 m neighborhood (blue circle).

We related our results regarding the proportions of species pairs with no association and segregation at 2 m and 10 m neighborhood to CBS plot, Sinharaja plot and BCI plot in Wiegand et al. [24] (Figure 3-a and b). The proportions of no assciation type at 2 m and 10 m neighborhood at BDGS plot did not fit the tendency that the proportion of no assciation increased with species richness and it had somewhat too low proportion of no assciation cases (Figure 3-a). The proprotion of segregation at 2 m neighborhood at BDGS plot fitted well with the tendency that the proportion of segregation decreased with speceis richness but that it failed in fitting the tendency at 10 m neighborhood (Figure 3-b).

### Analysis 2: Fine-scale Species Interactions

The GoF test detected for BDGS plot in 1,204 species pairs (47% of all cases) significantly departed from the Heterogeneous Poisson null model. 495 cases were negative (repulsion) and 709 cases were positive (attraction) (Table 1). The rank of the GoF test correlated strongly and positively with the product of the number of trees of the two species (r_sp_ = 0.484; P<0.01), the number of trees of the focal species (r_sp_ = 0.255; p<0.01) and the number of the second species (r_sp_ = 0.435; p<0.001) (Table S3). For species within the same family, positive fine-scale species interactions occurred more frequently (p<0.001) and negative ones occurred less frequently (p<0.001) than expected by chance (Table S4). For species within the same fruit type, positive fine-scale species interactions tended to occur more frequently and negative ones tended to occur less frequently but they were not significant with the 0.025 error rate.

**Table 1:** The proportion of species pairs significantly departed from Heterogeneous Poisson model and the proportion of no fine-scale species interactions cross life forms.

	All species	Arbor	Shrub	Arbor-Shrub	Deciduous	Evergreen	Deciduous - Evergreen
**Total pairs**	2550	930	380	1240	870	420	1260
**Proportion of no effect**	52.8%	34.0%	81.6%	58.1%	49.1%	56.9%	54.0%
**Proportion of attraction**	27.8%	49.2%	11.6%	16.7%	28.8%	24.8%	28.1%
**Proportion of repulsion**	19.4%	16.7%	6.8%	25.2%	22.1%	18.3%	17.9%
**Proportion of significant pairs**	47.2%	66.0%	18.4%	41.9%	50.9%	43.1%	46.0%

Goodness-of-Fit tests were used to test the overall fit of the Heterogeneous Poisson model over 0−30 m distance interval with the 0.05 error rate under 199 simulations; Arbor-shrub indicates the fine-scale species interaction between arbor species and shrub species; Deciduous-Evergreen indicates the fine-scale species interactions between deciduous species and evergreen species.

The proportion of significant fine-scale species interactions for arbor species (66%) was much higher than that for shrub species (18%) and the proportion of no association for shrub species (82%) was much higher than that for arbor species (34%) (Table 1). The proportion of significant fine-scale species interactions for deciduous species (51%) was slightly greater than that for evergreen species (43%) and the proportion of no association for deciduous species (49%) was a little smaller than that for evergreen species (51%) (Table 1).

**Figure 4:**
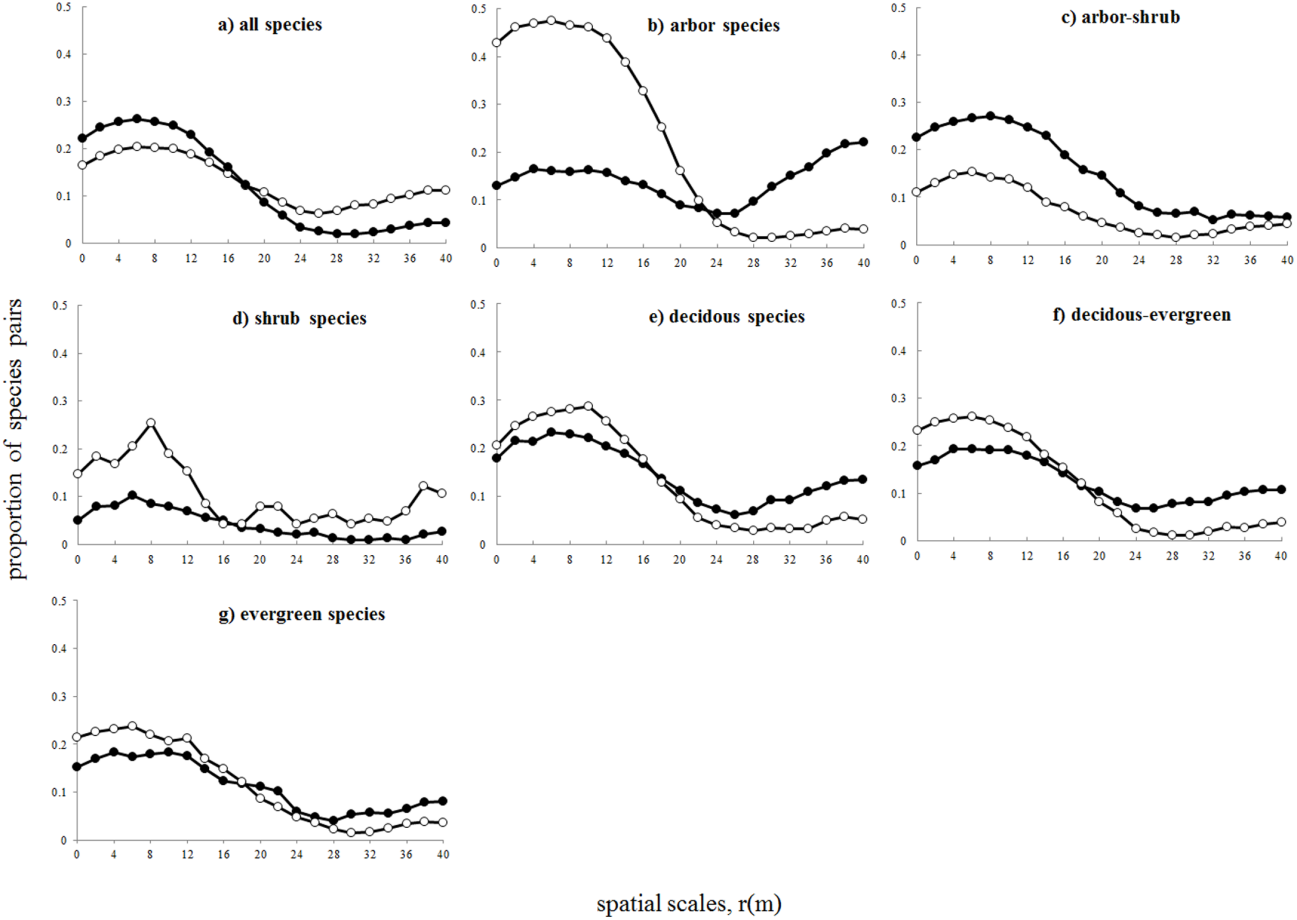
The proportion of significant interspecific associations for all species and different life forms at 0−50 m scale. a) All species, b) arbor species, c) shrub species, d) arbor-shrub species, e) deciduous species, f) evergreen species, g) deciduous-evergreen species.

We counted the number of species for which the pair-correlation function was above or below the simulation envelopes (Figure 4-a–g). Within fine-scales (0−15 m), the proportion of positive fine-scale species interactions always exceeded that of negative ones. Such trend was particularly obvious for arbor species. However, for shrub species, the positive fine-scale species interactions tended to be higher than negative ones (Figure 4-d) and for arbor-shrub species, the negative ones tended to be higher than positive ones (Figure 4-c). Figure 4 showed that the number of significant cases at larger scale (>30 m) were higher than the error rate of 0.05 (e.g., Figure. 4-a, b, c, e and g). This was a hint that the assumption of separation of scales was not fully met [6].

## Discussions

Determining how individuals of different species located with respected to each other is of basic importance for us to understand species assemblages, because the interspecific spacing of plants is closely linked to species coexistence [7], [24], [27], [28]. In this study, we performed a comprehensive spatial pattern analysis of overall species associations and fine-scale species interactions at a fully-mapped 25-ha FDP in a species-rich subtropical forest at BDGS in central China. Most importantly, we found that nearly one half of all species pairs (47%) at BDGS plot showed significant fine-scale species interactions and it produced more positive ones than the negative ones. Positive ones occurred more frequently than expected by chance between species which shared one family and negative ones occurred less frequently than expected.

### Analysis of Overall Species Associations

In species-rich communities, encounters between heterspecific pairs would be rare. The stochastic dilution effect would make the overall species association less detectable [6], [24]. In the overall pattern analysis (analysis 1) using Homogeneous Poisson processes as a null model, we found that the no association type dominated the species associations until 8 m neighborhood. At neighborhoods larger than 20 m, we found that the overall species association patterns were dominated by segregation and partial overlap (greater than 70%) which suggested species tended to avoid each other at these scales. In addition, the proportion of the positive associations (i.e., mixing type III) was relative high at 6 m neighborhood, but they completely disappeared at 30 m neighborhood. All these suggested strong effects of habitat heterogeneity at larger scales. It probably was caused by the strong topographic structuring similar to that at the Sinharaja plot [24].

We also related our results regarding the proportion of species pairs with no association and with segregation at 2 m and 10 m scales to other forests (CBS plot, Sinharaja plot and BCI plot) in Wiegand et al. [24]. As expected, dilution effects at BDGS plot was stronger than at CBS plot and was weaker than BCI plot. However, contrary with hypothesis H1, we found that it had somewhat too low proportion of no association cases at BDGS plot. Even at fine scale (e.g., 6 m), the no association type at BDGS plot was 41% which was also lower than that at Sinharaja plot (58%; 205 species). For segregation at 10 m neighborhood, it failed again in fitting the tendency that segregation decreased with species richness which was against with hypothesis H2. But for segregation at 2 m neighborhood, it fitted well with the tendency. This was in accordance with hypothesis H2. For the overall species association analysis, our result partly supported the stochastic dilution hypothesis. Species richness at BDGS plot was much higher than at CBS plot and was much lower than at BCI plot. In contrast, the species richness at BDGS plot and at Sinharaja plot was relatively similar, but the topographic structure at BDGS plot with deep valleys and steep slopes was stronger than Sinharaja plot. Perhaps, the strong topographic structure at BDGS plot counteracted the signal of stochastic dilution effects in the overall species association patterns.

### Fine-scale Species Interactions

The stochastic dilution hypothesis assumes that the stochastic dilution effect could overpower the species interactions in species-richness communities. We found that a large proportion of the species pairs (47%) showed significant fine-scale species interactions (analysis 2) in this species-rich forest. Such high proportion of significant fine-scale species interactions here was quite different from previous studies conducted at other species-rich forests. For example, Wiegand et al. [6], [24] found that only a low proportion of species pairs (about 5%) showed significant species interactions in Sinharaja plot and Barro Colorado Island plot. Volkov et al. [26] also found that the interspecific interactions between 20 most common species were quite weak compared with intraspecific interactions. However, we should note that such strong fine-scale species interactions at BDGS plot may not indicate strong ‘real’ species interaction between species pairs but also reflected the effects of fine-scale environment filtering. In this study, we used the HP processes as the null model that can approximately factor out the effects of environment and therefor reveal potential effects of species interaction [6]. The assumption of this method is that environment effects on species vary over spatial scales which are larger than the bandwidth of the kernel estimate (30 m in this study). It is well-performed in many species interaction studies conducted at forest plots with the relative weak topographic structure e.g., 25-ha CBS plot [7], [24] and 50-ha BCI plot [24] or with a strong large-scale topographic structure e.g., 25-ha Sinharaja plot [6], [24]. Differently, our study plot showed a very strong fine-scale topographic structure with deep valleys and steep slopes (Figure S1). Therefore, the habitat suitability for species changes abruptly at scales of tens of meters here (e.g., Figure S2-a and c). This would make the assumption of separation of scales invalid and we cannot separate the effects of environment and species interaction using this method. In addition, the number of significant species pairs was higher than the 0.05 error rate in about and beyond 30 m scales. This was also a hint that the assumption of separation of scales was not fully met in this forest. On the other hand, if high incidence of fine-scale effects reflected the ‘real’ species interaction, one can predict that species within similar properties should compete more strongly [16]. However, we found that the positive fine-scale species interactions occurred more frequently and negative ones occurred less frequently for species within the same family than expected by chance. This was in accordance with the prediction of environmental filtering that species with similar ecological requirement co-occurred in the local habitats [16], [18], [19]. However, we should conclude cautiously as this result could also arise from competitive exclusion according to recent results of coexistence theory (e.g., Mayfield and Levine [17]). To better reveal species interactions in this plot, we would need a more detailed assessment of the intensity function based on the explicit environmental covariates (e.g., topographic factor and soil factors) [39].

### The Influence of the Life Form on Observed Fine-scale Species Interactions

There is a large number of species (238) at the 25-ha BDGS plot which is even higher than that (207) at the equal sized tropical forest in Sinharaja, Sri Lanka [6], [24]. Compared with tropical forests, one of remarkable features in community structure for BDGS forests is that it is rich in shrub species in the vegetation layer below 5 m. In other words, there are less species which could reach the arbor layer at BDGS plot than that at tropical forests. Previous studies conducted at BCI plot found that arbor-arbor species interactions were stronger than the understory-understory species interaction [26]. We found that the proportion of significant fine-scale species interactions for arbor species (66%) was much higher than that for shrub species (18%) and the proportion of no effects for arbor species (34%) was much lower than that for shrub species (82%). However, we should note that the assumption of separation of scale for analysis 2 was not fully met in this study and results of the analysis showed the effect of environment. Possibly, this may suggest that arbor species experienced stronger environmental filtering than shrub species. We found that the adult trees of arbor species tended to be more patchily distributed than those of shrub species from the species distribution maps (Figure S2-a, c, e and g). Another analysis in the 25-ha BDGS plot also showed that the degree of topographic structuring for arbor species was significantly stronger than that for shrub species (Wang et al. Unpublished data). Another possible explanation for that arbor species showed such a high incidence of fine-scale species interactions compared with shrub species was abundance effects. As the number of shrub trees with DBH over 10 cm were generally less than those of arbor trees (Figure S3). Because the power of the GoF test was a function of abundance, the significant fine-scale effects would be more likely detected when the species were more abundant [42]. Our result showed that the significant fine-scale species interactions were more likely to happen for more abundant species.

## Conclusions

The spatial patterns of larger trees are the outcome of different processes and mechanisms during regeneration and growth. We analyzed the 2,550 overall species associations and fine-scale species interactions at a 25-ha forest plot. Combined with results of overall species association pattern from other study [24], we found that it partly supported the stochastic dilution hypothesis. On the other hand, we obtained a high proportion of significant species pairs in the fine-scale species interaction analyses. However, the study plot performed a strong fine-scale topographic structure and the assumption of separation of scales was not fully met. With the method we used, we cannot reveal the effects of species interaction in this study plot. To separate the effects of environment and species interaction in our plot with the strong fine-scale environment heterogeneity, we should further assess the intensity function using explicit environmental covariates.

## Supporting Information

Figure S1 **Topographic map in 25-ha Badagongshan Forest Dynamic plot (after Wang et al. unpublished data).**
(DOCX)

Figure S2 **Example for analysis of the fine-scale species interaction for species pairs of arbor and shrub.**
(DOCX)

Figure S3 **Species abundance distribution for 31 arbor species and 20 shrub species in this study.**
(DOCX)

Table S1 **Species properties for 51 species in this study.**
(DOCX)

Table S2 **Summary of the results of fine-scale species interactions between species pairs.** The lines give the focal species and the columns species 2. Given is the rank of the Goodness-of Fit test for significant cases and the sign indicates positive or negative effects with a p-value<0.05. For species codes see Appendix A1.
(XLSX)

Table S3 **Correlation of the rank of Goodness-of-Fit test of fine-scale species association analysis with the several variables representing univariate species aggregation and species abundance.** The correlations are not corrected for multiple testing. *n*_sp1_: number of individuals of species 1, *n*_sp2_: number of individuals of species 2, *g*_11_ and *g*_22_ are the values of the univariate pair correlation functions of species 1 and 2, respectively, at the specified spatial scale *r*. *P<0.05, **P<0.01; ***P<0.001.
(DOCX)

Table S4 **Permutation analysis of the association matrix shown in Table S2 to find out if species with shared family and fruit types show more or less significant associations than expected by random distribution of significant cases over all pairs of species.** *n*_t_: total number of significant associations of specified type; *m*_t_: total number of pairs with shared family or fruit type; *n*_s_: number of pairs with shared family or fruit type which show a significant association; *n*_s_^exp^: expectation of *n*_s_ under 10,000 randomization of significant cases over all pairs of species.
(DOCX)
