# Supplementary material for: Species Associations in a Species-Rich Subtropical Forest Were Not Well-Explained by Stochastic Geometry of Biodiversity
Source: PLoS One. 2014 May 13;9(5):e97300. doi: 10.1371/journal.pone.0097300 (PMC4019537; doi:10.1371/journal.pone.0097300)
Supplement: Figure S1 — Topographic map in 25-ha Badagongshan Forest Dynamic plot (after Wang et al. unpublished data). (DOCX) [file pone.0097300.s001.docx]

Fig S1 topographic map in 25-ha Badagongshan Forest Dynamic plot (after Wang et al. unpublished data). (DOCX)
