# Supplementary material for: Species Associations in a Species-Rich Subtropical Forest Were Not Well-Explained by Stochastic Geometry of Biodiversity
Source: PLoS One. 2014 May 13;9(5):e97300. doi: 10.1371/journal.pone.0097300 (PMC4019537; doi:10.1371/journal.pone.0097300)
Supplement: Figure S2 — Example for analysis of the fine-scale species interaction for species pairs of arbor and shrub. (DOCX) [file pone.0097300.s002.docx]

| Arbor species | |
| --- | --- |
| 1. *Rhododendron stamineum* (red circle) and *Enkianthus serrulatus* (blue circle) | 1. *Rhododendron stamineum* VS. *Enkianthus serrulatus* |
| 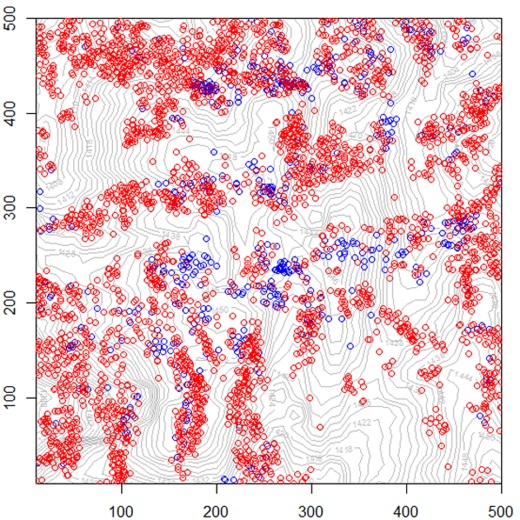 | 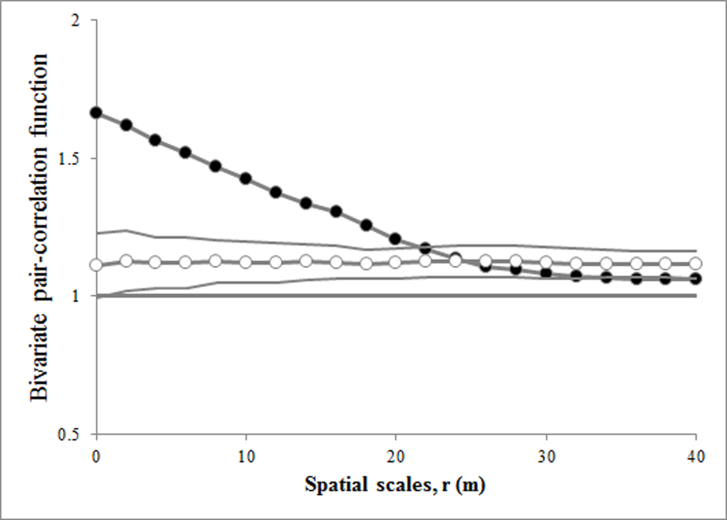 |
| c) *Machilus ichangensis* (red circle) and *Quercus serrate* (blue circle) | 1. *Machilus ichangensis* VS. *Quercus serrate* |
| 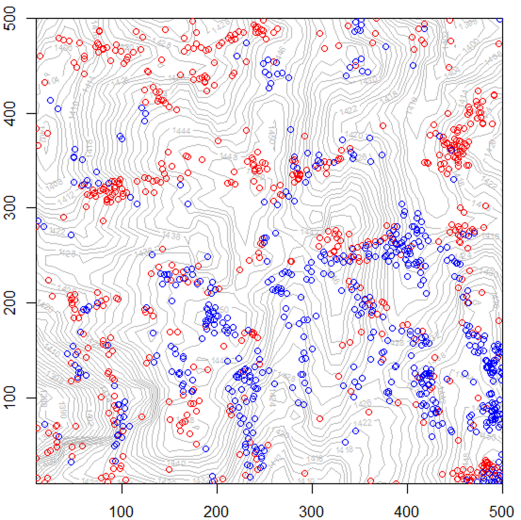 | 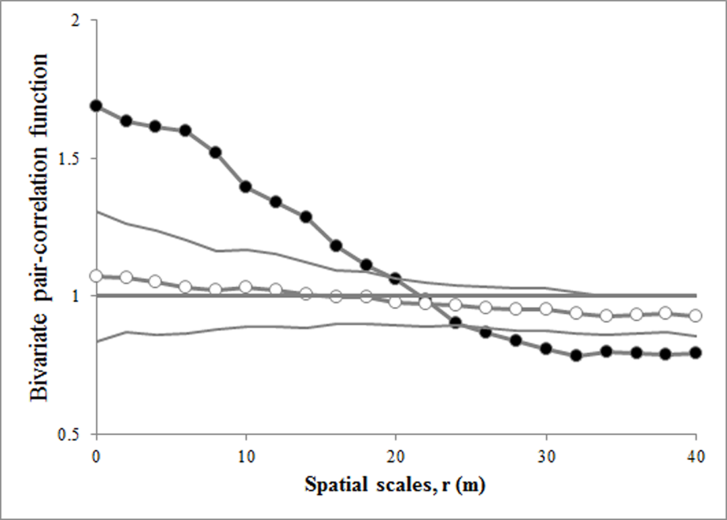 |
| Shrub species | |
| 1. *Lindera erythrocarpa* (red circle) and *Photinia beauverdiana* (blue circle) | 1. *Lindera erythrocarpa* VS. *Photinia beauverdiana* |
| 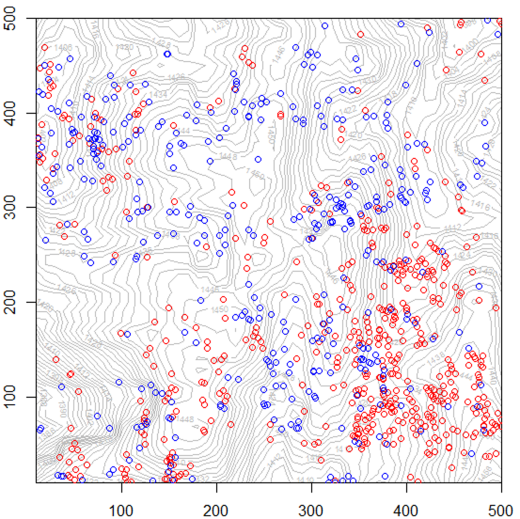 | 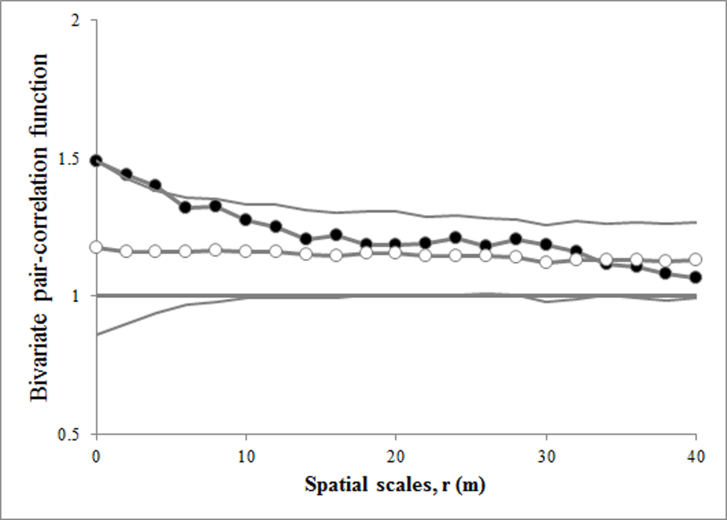 |
| g) *Neolitsea aurata var. paraciculata* (red circle) and *Cornus elliptica* (blue circle) | (h) *Neolitsea aurata var. paraciculata* VS. *Cornus elliptica* |
| 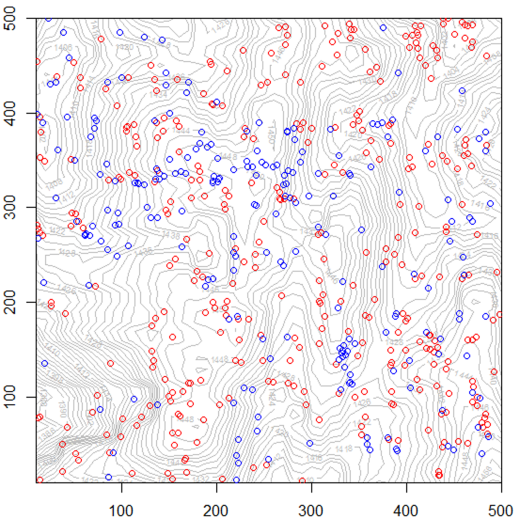 | 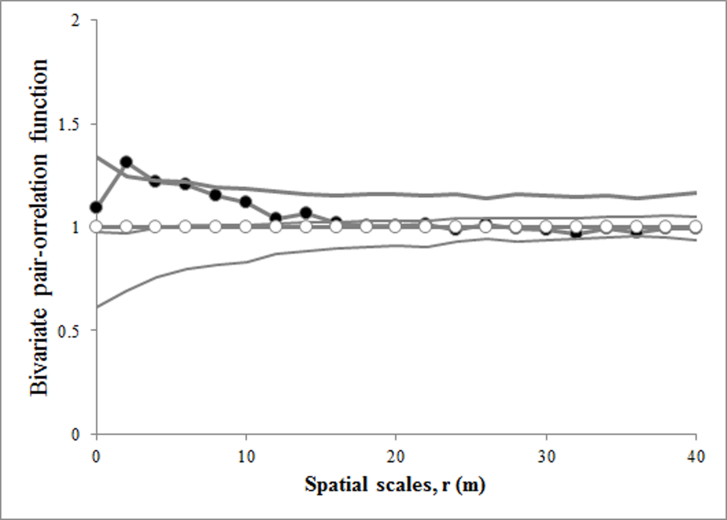 |

**Fig S2. Examples of spatial distributions and bivariate fine-scale species interactions for arbor species (a-d) and shrub species (e-h)**. Shown are the bivariate g_12_ pair-correlation function of the data in dependence on scale r (closed circle), the expected g_12_ function under the heterogeneous Poisson null model (open circle) and the simulation envelopes (gray line) being the fifth-lowest and the fifth-highest values of the Monte Carlo simulations of the null modes. The horizontal lines give the expected g_12_ function for independent patterns.
