# Supplementary figures and images for: Species Associations in a Species-Rich Subtropical Forest Were Not Well-Explained by Stochastic Geometry of Biodiversity
Source: PLoS One. 2014 May 13;9(5):e97300. doi: 10.1371/journal.pone.0097300 (PMC4019537; doi:10.1371/journal.pone.0097300)

| 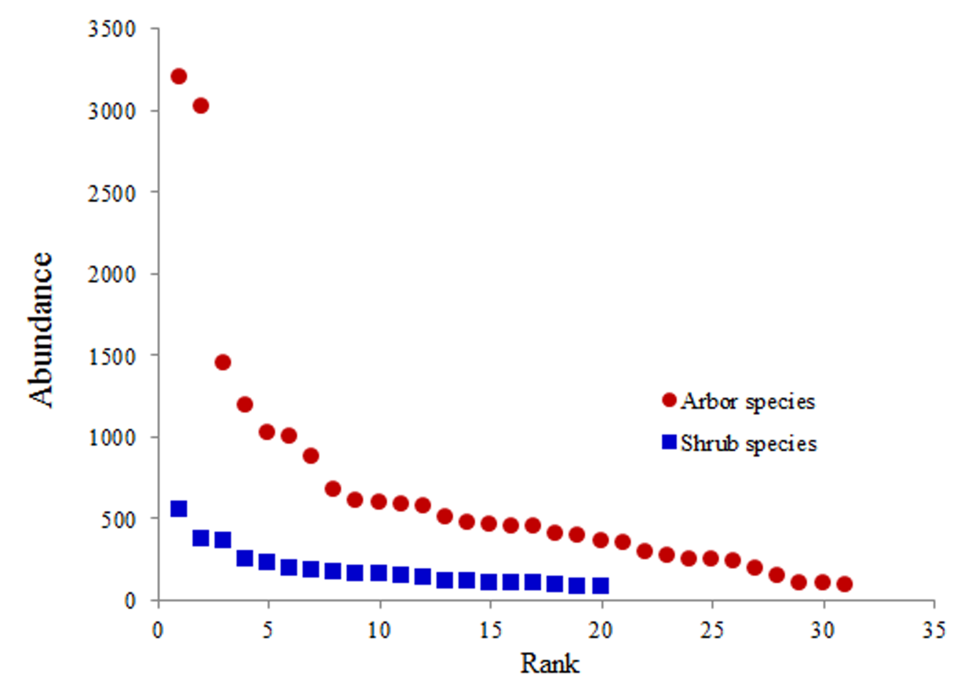 |
| --- |

Figure S3 Species abundance distribution for 31 arbor species and 20 shrub species in this study.

Supplement: Figure S3 — Species abundance distribution for 31 arbor species and 20 shrub species in this study. (DOCX) [file pone.0097300.s003.docx]
