# Supplementary material for: Species Associations in a Species-Rich Subtropical Forest Were Not Well-Explained by Stochastic Geometry of Biodiversity
Source: PLoS One. 2014 May 13;9(5):e97300. doi: 10.1371/journal.pone.0097300 (PMC4019537; doi:10.1371/journal.pone.0097300)
Supplement: Table S1 — Species properties for 51 species in this study. (DOCX) [file pone.0097300.s004.docx]

Table S1 Species properties for 51 species in this study

|  | species | code | life form | life form | family | count | fruit type |
| --- | --- | --- | --- | --- | --- | --- | --- |
| 1 | *Rhododendron stamineum* | RHOSTA | Arbor | Evergreen species | Ericaceae | 3195 | Capsule |
| 2 | *Cyclobalanopsis multinervis* | CYCMUL | Arbor | Evergreen species | Fagaceae | 3021 | Nut |
| 3 | *Cornus kousa* | CORKOU | Arbor | Deciduous species | Cornaceae | 1450 | Sorosis |
| 4 | *Fagus lucida* | FAGLUC | Arbor | Deciduous species | Fagaceae | 1188 | Nut |
| 5 | *Carpinus fargesii* | CARFAR | Arbor | Deciduous species | Betulaceae | 1025 | Nut |
| 6 | *Rhododendron fortunei* | RHOFOR | Arbor | Evergreen species | Ericaceae | 997 | Nut |
| 7 | *Cyclobalanopsis gracilis* | CYCGRA | Arbor | Evergreen species | Fagaceae | 877 | Nut |
| 8 | *Lyonia ovalifolia* | LYOOVA | Arbor | Deciduous species | Ericaceae | 671 | Capsule |
| 9 | *Machilus ichangensis* | MACICH | Arbor | Evergreen species | Lauraceae | 606 | Drupe |
| 10 | *Quercus serrata* | QUESER | Arbor | Deciduous species | Fagaceae | 597 | Nut |
| 11 | *Nyssa sinensis* | NYSSIN | Arbor | Deciduous species | Nyssaceae | 588 | Drupe |
| 12 | *Enkianthus serrulatus* | ENKSER | Arbor | Deciduous species | Ericaceae | 572 | Capsule |
| 13 | *Lindera erythrocarpa* | LINERY | Shrub | Deciduous species | Lauraceae | 549 | Drupe |
| 14 | *Schima parviflora* | SCHSUP | Arbor | Evergreen species | Theaceae | 508 | Capsule |
| 15 | *Toxicodendron succedaneum* | TOXSUC | Arbor | Deciduous species | Anacardiaceae | 466 | Drupe |
| 16 | *Sorbus folgneri* | SORFOL | Arbor | Deciduous species | Rosaceae | 462 | Pome |
| 17 | *Meliosma oldhamii* | MELOLD | Arbor | Deciduous species | Sabiaceae | 447 | Drupe |
| 18 | *Castanea seguinii* | CASSEG | Arbor | Deciduous species | Fagaceae | 446 | Nut |
| 19 | *Betula insignis* | BETINS | Arbor | Deciduous species | Betulaceae | 406 | Nut |
| 20 | *Sassafras tzumu* | SASTZU | Arbor | Deciduous species | Lauraceae | 393 | Drupe |
| 21 | *Photinia beauverdiana* | PHOBEA | Shrub | Deciduous species | Rosaceae | 373 | Berry |
| 22 | *Neolitsea aurata var. paraciculata* | LINOBT | Shrub | Deciduous species | Lauraceae | 359 | Drupe |
| 23 | *Sorbus wilsoniana* | SORWIL | Arbor | Deciduous species | Rosaceae | 357 | Pome |
| 24 | *Litsea elongata* | LITELO | Arbor | Evergreen species | Lauraceae | 349 | Drupe |
| 25 | *Symplocos macrophylla* | SYMMAC | Arbor | Evergreen species | symplocaceae | 293 | Drupe |
| 26 | *Ilex suaveolens* | ILESUA | Arbor | Evergreen species | Aquifoliaceae | 264 | Drupe |
| 27 | *Liquidambar acalycina* | LIQACA | Arbor | Deciduous species | Hamamelidaceae | 250 | Capsule |
| 28 | *Symplocos groffii* | SYMGRO | Arbor | Evergreen species | symplocaceae | 250 | Drupe |
| 29 | *Camellia pitardii* | CAMPIT | Shrub | Evergreen species | Theaceae | 241 | Capsule |
| 30 | *Quercus engleriana* | QUEENG | Arbor | Evergreen species | Fagaceae | 233 | Nut |
| 31 | *Cornus elliptica* | CORELL | Shrub | Evergreen species | Cornaceae | 222 | Sorosis |
| 32 | *Cornus controversa* | CORCON | Arbor | Deciduous species | Cornaceae | 187 | Drupe |
| 33 | *Padus grayana* | PADGRA | Shrub | Deciduous species | Rosaceae | 186 | Drupe |
| 34 | *Rhododendron mariesii* | RHOMAR | Shrub | Deciduous species | Ericaceae | 182 | Capsule |
| 35 | *Symplocos pseudobarberina* | SYMPSE | Shrub | Evergreen species | symplocaceae | 162 | Drupe |
| 36 | *Symplocos lucida* | SYMLUC | Shrub | Evergreen species | symplocaceae | 160 | Drupe |
| 37 | *Euonymus myrianthus* | EUOMYR | Shrub | Evergreen species | Celastraceae | 158 | Capsule |
| 38 | *Acer davidii* | ACEDAV | Arbor | Deciduous species | Aceraceae | 150 | Samara |
| 39 | *Weigela japonica* | RHOSTA | Shrub | Deciduous species | Caprifoliaceae | 144 | Drupe |
|  |  |  |  | To be continued |  |  |  |
|  |  |  |  | Continued |  |  |  |
| 40 | *Hydrangea strigosa* | WEIJAP | Shrub | Deciduous species | Saxifragaceae | 128 | Capsule |
| 41 | *Euscaphis japonica* | HYDSTR | Shrub | Deciduous species | Staphyleaceae | 114 | Follicle |
| 42 | *Eurya brevistyla* | EUSJAP | Shrub | Evergreen species | Theaceae | 113 | Berry |
| 43 | *Symplocos anomala* | EURBRE | Shrub | Evergreen species | symplocaceae | 105 | Drupe |
| 44 | *Clethra fargesii* | SYMANO | Arbor | Deciduous species | Clethraceae | 104 | Capsule |
| 45 | *Enkianthus chinensis* | CLEFAR | Shrub | Deciduous species | Ericaceae | 104 | Capsule |
| 46 | *Vaccinium bracteatum* | ENKCHI | Arbor | Evergreen species | Ericaceae | 96 | Berry |
| 47 | *Acer oliverianum* | VACBRA | Shrub | Deciduous species | Aceraceae | 95 | Samara |
| 48 | *Daphniphyllum macropodum* | ACEOLI | Shrub | Evergreen species | Daphniphyllaceae | 93 | Drupe |
| 49 | *Carpinus turczaninowii* | DAPMAC | Arbor | Deciduous species | Betulaceae | 89 | Nut |
| 50 | *Litsea veitchiana* | CARTUR | Shrub | Deciduous species | Lauraceae | 77 | Drupe |
| 51 | *Camellia cuspidata* | LITVEI | Shrub | Evergreen species | Theaceae | 76 | Capsule |
