# Supplementary material for: Species Associations in a Species-Rich Subtropical Forest Were Not Well-Explained by Stochastic Geometry of Biodiversity
Source: PLoS One. 2014 May 13;9(5):e97300. doi: 10.1371/journal.pone.0097300 (PMC4019537; doi:10.1371/journal.pone.0097300)
Supplement: Table S3 — Correlation of the rank of Goodness-of-Fit test of fine-scale species association analysis with the several variables representing univariate species aggregation and species abundance. The correlations are not corrected for multiple testing. n sp1: number of individuals of species 1, n sp2: number of individuals of species 2, g 11 and g 22 are the values of the univariate pair correlation functions of species 1 and 2, respectively, at the specified spatial scale r. *P<0.05, **P<0.01; ***P<0.001. (DOCX) [file pone.0097300.s006.docx]

**Table S3 Correlation of the rank of Goodness-of-Fit test of fine-scale species association analysis with the several variables** [**represent**](app:ds:represent)**ing univariate species aggregation and species abundance.** The correlations are not corrected for multiple testing. *n*_sp1_: number of individuals of species 1, *n*_sp2_: number of individuals of species 2, *g*_11_ and *g*_22_ are the values of the univariate pair correlation functions of species 1 and 2, respectively, at the specified spatial scale *r*. * P < 0.05, ** P < 0.01; ***P < 0.001.

| Variable | Correlation *r*_SP_ |
| --- | --- |
| *n*_sp1_ × *n*_sp2_ | 0.484*** |
| *n*_sp1_ + *n*_sp2_ | 0.467*** |
| *n*_sp1_ | 0.255*** |
| *n*_sp2_ | 0.435*** |
| *g*_11_(*r*= 0m) | -0.120** |
| *g*_11_(*r*= 6m) | -0.131*** |
| *g*_11_(*r*= 10m) | -0.122*** |
| *g*_11_(*r*= 20m) | -0.135*** |
| *g*_11_(*r*= 30m) | -0.201*** |
| *g*_22_(*r*= 0m) | -0.120*** |
| *g*_22_(*r*= 6m) | -0.129*** |
| *g*_22_(*r*= 10m) | -0.122*** |
| *g*_22_(*r*= 20m) | -0.135*** |
| *g*_22_(*r*= 30m) | -0.201*** |
