# Supplementary material for: Species Associations in a Species-Rich Subtropical Forest Were Not Well-Explained by Stochastic Geometry of Biodiversity
Source: PLoS One. 2014 May 13;9(5):e97300. doi: 10.1371/journal.pone.0097300 (PMC4019537; doi:10.1371/journal.pone.0097300)
Supplement: Table S4 — Permutation analysis of the association matrix shown in Table S2 to find out if species with shared family and fruit types show more or less significant associations than expected by random distribution of significant cases over all pairs of species. n t: total number of significant associations of specified type; m t: total number of pairs with shared family or fruit type; n s: number of pairs with shared family or fruit type which show a significant association; n s exp: expectation of n s under 10,000 randomization of significant cases over all pairs of species. (DOCX) [file pone.0097300.s007.docx]

**Table S4 Permutation analysis of the association matrix shown in Table S2 to find out if species with shared family and fruit types show more or less significant associations than expected by random distribution of significant cases over all pairs of species.** *n*_t_ : total number of significant associations of specified type; *m*_t_ : total number of pairs with shared family or fruit type; *n*_s_ : number of pairs with shared family or fruit type which show a significant association; *n*_s_^exp^ : expectation of *n*_s_  under 10000 randomization of significant cases over all pairs of species.

|  | Associations types | *n*_t_ | *m*_t_ | *n*_s_ | *n*_s_^exp^ | P-value |
| --- | --- | --- | --- | --- | --- | --- |
| Family | Attraction | 709 | 160 | 67 | 44.4 | <0.001 |
|  | Repulsion | 495 | 160 | 14 | 31.1 | <0.001 |
| Fruit type | Attraction | 709 | 576 | 163 | 160.2 | 0.406 |
|  | Repulsion | 495 | 576 | 96 | 111.8 | 0.030 |
